# Titania‐Based Coral‐Structured Solar Absorber Coating with Improved Scalability and Durability at High Temperature

**DOI:** 10.1002/advs.202407409

**Published:** 2024-09-20

**Authors:** Yifan Guo, Kaoru Tsuda, Milad Mohsenzadeh, Sahar Hosseini, Yasushi Murakami, Joe Coventry, Juan F. Torres

**Affiliations:** ^1^ ANU HEAT Lab School of Engineering The Australian National University Canberra 2601 Australia; ^2^ Nano Frontier Technology Tokyo 141‐0032 Japan; ^3^ Thermal Energy Group School of Engineering The Australian National University Canberra 2601 Australia; ^4^ Faculty of Textile Science and Technology Shinshu University Ueda 386‐8567 Japan

**Keywords:** concentrating solar power, hierarchical structure, materials degradation, solar energy harvesting, solar energy materials

## Abstract

Solar energy harvesting and storage are essential in the future mix of renewable energy technologies. Hierarchical coral‐structured coatings have been shown to yield high solar absorptance in concentrating solar thermal (CST) systems. However, interfacial delamination and scalability challenges owing to material complexity pose significant hurdles for the widespread industrial adoption of these hierarchical CST coatings. Here, a coral‐structured coating is proposed whose black pigments are strongly bonded by titania, which is a material that mitigates interfacial delamination. Importantly, this coating follows a facile deposition procedure suitable for large‐scale solar receivers. The drone‐deposited coating inhibits cation diffusion and maintains a stable solar absorptance of 97.39±0.20% even after long‐term (3000 h) high‐temperature (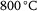) aging. The scalability of developed coating represents a substantial advancement in the implementation of light‐trapping enhancement and maintenance approaches across a wide range of CST applications.

## Graphical abstract

A coral‐structured coating, whose black pigments are strongly bonded by titania, is proposed. The coating inhibits cation diffusion and maintains a stable solar absorptance of 97.39±0.20% even after long‐term high‐temperature aging. The scalability of the coating with a facile deposition procedure represents a substantial advancement in the implementation of light‐trapping enhancement and maintenance approaches for high‐temperature solar harvesting technologies.

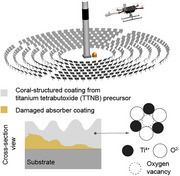

## 1 Introduction

A solar thermal receiver is an essential component of concentrating solar thermal (CST) systems, as it converts concentrated sunlight into heat. Conventional receivers are always coated with a solar absorber coating, aiming to have 100% absorption, such as Pyromark.^[^ ^1^, ^2^ ^]^ Although the spectral selectivity of the coating absorptance is considered by many studies, the absorptance has a much higher impact than the emittance on the overall thermal efficiency of a high‐flux, high‐temperature CST receiver.^[^ ^3^, ^4^ ^]^ In addition, durability is another factor to determine the performance of solar absorber coatings because CST plants need to operate for decades and these CST coatings are generally not easily accessible for maintenance, as they are usually placed 200 m above ground.^[^ ^5^ ^]^ Light‐trapping morphologies with nanopores extended throughout the entire coating volume have been the general approach in CST.^[^ ^6^, ^7^, ^8^ ^]^ However, solar absorber coatings tend to deteriorate in optical performance and even suffer physical damage such as delamination during long‐time operation.^[^ ^9^ ^]^ A groundbreaking absorber coating with coral‐structured features was developed to solve such a problem.^[^ ^10^ ^]^ The unique feature of the coating is its multi‐scale hierarchical architecture, which includes an antireflective nanolayer.^[^ ^11^ ^]^ To the best of our knowledge, the coating exhibits the highest absorptance of any solar absorber coating currently available at such an elevated temperature.

**Figure 1:**
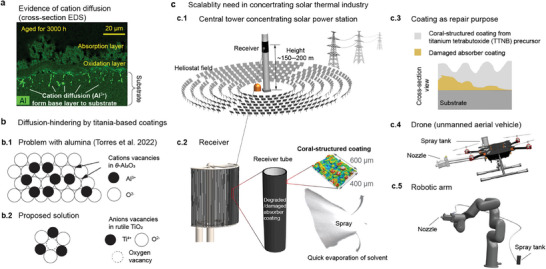
Concept of scalable coral‐structured coating with potential absence of cation diffusion. a) EDS images showing cation (Al^3+^) diffusion in the previous study.^[^ ^10^ ^]^ b) Schematic diagram of the crystal structure of two compounds. b.1) θ‐Al_2_O_3_ with cations vacancies, indicating the previous problem and b.2) Rutile TiO_2_ with oxygen vacancy for the proposed solution. The newly developed coral‐structured coating that has titania as a binder in its base layer. We hypothesize that, due to the kinetics of the cation Ti^4+^ in the coating at high temperatures, it is unlikely that cation diffusion occurs, improving the durability of the coating. c) Concept of coatings scalability in an industrial, large‐scale concentrating solar thermal (CST) plant (normally >10^3^ km^2^ in land area). c.1) The potential application of the coating proposed in this study for central solar thermal receivers in towers as high as 150–200 m. These receivers can be as large as 30 m in height. c.2) Diagram showing the coral‐structured coating on degraded or damaged solar absorber coatings as means of maintenance or repair, respectively. c.3) Diagram showing the cross‐section view after depositing the proposed coral‐structured coating on the damaged Pyromark as a means of repairing the solar‐absorbing surface. c.4) Drone‐based deposition of coral‐structured coating. c.5) Robotic arm‐based deposition. In this study, both scalability approaches are tested.

Although it is the best‐performing coating with solar absorption so far, the coral‐structured coating still exhibits some durability issues. The ability of the coating to resist absorptance changes even after experiencing changes in morphology at specific length scales could vary during the aging process. The antireflective layer, which contains silica nanoparticles located at the top of the coating, was shown to be stable in our previous study.^[^ ^11^ ^]^ In our previous works,^[^ ^10^ ^]^ alumina was used in the base layer as an inorganic binder between black spinel pigments because it provided a good bonding strength between the base layer of the coating and its metallic substrate (nickel‐based alloy). However, for extensive aging exceeding 3000 h, the base layer disappeared, diffusing through the voids in the metallic substrate, as shown by the energy‐dispersive X‐ray spectroscopy (EDS) analysis in **Figure** **1**a. Cation (Al^3+^) deposition within the substrate was observed, which is an indication of cation diffusion. The only source of Al^3+^ cations is the alumina (Al_2_O_3_) in the base layer, which was introduced by the precursor of the aluminum complex (aluminum ethylaceto acetate di isopropirate, or S75P). Different phases of alumina can be obtained with a gradual increase in temperature when heating alumina.^[^ ^12^, ^13^, ^14^ ^]^ Before reaching the stable phase α‐Al_2_O_3_ at around 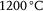, some meta‐stable transition phases are formed, such as γ, δ and θ. Evolution of γ‐Al_2_O_3_ (being stable below 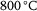) to δ‐ or θ‐Al_2_O_3_ can proceed during the temperature range of 750–1000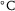.^[^ ^13^, ^15^ ^]^ Regardless of the transition phase, cation (Al^3+^) vacancies always exist in the alumina lattice,^[^ ^16^ ^]^ as shown in Figure 1b.1. With a cubic‐closed‐packed stack, vacancies are on both the octahedral and tetrahedral sites for γ‐ and θ‐Al_2_O_3_.^[^ ^17^ ^]^ This also contributes to the structure disorder in the compound lattice.^[^ ^17^, ^18^ ^]^ Cation diffusion is more likely to occur and the observed Al^3+^ penetration in Figure 1a may lead to interfacial delamination between the coating and its substrate. This degradation mechanism may lead to the known failure of spallation and delamination.^[^ ^8^ ^]^

Scalability is a requirement for newly developed solar absorber coatings, both in materials preparation and coating deposition, because conventional receivers are generally larger than 10 m.^[^ ^19^ ^]^ In industrial settings, scaling up coating processes onto large surfaces presents significant challenges, particularly in terms of safety hazards for operators and maintaining process control and repeatability. We developed a coating method using a drone^[^ ^20^ ^]^ and a robotic arm to address these challenges, focusing on the scalability and characterization of coatings on large preheated surfaces. The deposition of coatings via the robotic arm is a standard approach in the industry, commonly used in various applications such as car manufacturing^[^ ^21^ ^]^ and medical treatment.^[^ ^22^ ^]^ In addition, when increasing the maturity of a technology from a low technology readiness level (TRL) to a high TRL, several challenges often arise. One prevalent issue is the complexity of the material architecture. The original coral‐structured coating reported in ref. [10] was developed from three different inorganic binders (alumina for the base layer, titania for the absorption layer and silica for the nanolayer), while a single binder is used in mature technologies such as Pyromark 2500^[^ ^8^ ^]^ and in newly developed nanoporous coatings.^[^ ^23^ ^]^ Furthermore, the thermal expansion mismatch between the base layer and its substrate has an impact on thermal stress^[^ ^8^, ^24^ ^]^ and, hence, mechanical durability. Moreover, previously the coral‐structured coating was formed by spray deposition with a very large number of passes (>20), which could lead to overgassing, i.e., the deposition of the inorganic binder on adjacent regions. Overgassing negatively impacts the solar absorptance of the coating. Additionally, CST coatings generally degraded or damaged^[^ ^9^, ^25^ ^]^ after extensive service, so maintenance and repair with minimal interruption to daily operation of the power plant are needed. As the coral‐structured architecture is formed when the substrate is kept at elevated temperatures of up to 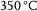,^[^ ^10^ ^]^ a receiver at these high temperatures may pose safety risks to the operator who performs the re‐application if done manually.^[^ ^26^ ^]^ Furthermore, the need for frequent re‐application^[^ ^5^ ^]^ on high receivers increases costs. Addressing these numerous technical challenges requires a comprehensive approach that balances coating quality (optical and mechanical), safety considerations, and cost‐effectiveness.

Here, we propose and evaluate a coral‐structured coating with titania as an inorganic binder in the base and absorption layers. Titania not only provides strong adhesion between the substrate and coating but also acts as a means of hindering cation diffusion to the substrate, which is expected to improve durability. In contrast to the alumina lattice that contains cation vacancies, most of the deficiency in rutile titania (TiO_2_) is oxygen anions,^[^ ^27^, ^28^, ^29^ ^]^ as shown in Figure 1b. With a potential for oxygen adsorption, titanium has a low diffusion rate that may inhibit the penetration of cations into the substrate. This could further improve the durability of coral‐structured coatings. Moreover, the proposed coating uses the same material (precursor) for the base and absorption layers. Streamlining the materials preparation and deposition process can significantly improve scalability. Exploring cost‐effective deposition methods for the coral‐structured coatings and their re‐application on receivers (Figure 1c.1) is paramount for cost efficiency. Our proposed coating deposition method is suitable for in situ maintenance of degraded absorbers or for the repair of damaged coatings. The proposed coral‐structured coating can cover the defective part of the receiver tube with a simple spray deposition method, as shown in Figure 1c.2. Regardless of the coating substrate (either a pristine metallic tube or coated with a degraded coating), our proposed coating can be applied effectively, as shown in Figure 1c.3. Importantly, we further evaluated the scalability in an industrial setting. The practical feasibility and performance of coral‐structured coatings on a large scale is validated with a drone (unmanned aerial vehicle, Figure 1c.4) and a robotic arm (Figure 1c.5), paving the way for the widespread adoption of coral‐structured coatings in CST.

## 2 Results

### 2.1 Evaluation of Different Precursors

The coral‐structured coating is a recently developed light‐trapping architecture with hierarchical morphology inspired by the natural formation of stony coral.^[^ ^10^ ^]^ These coatings are designed to efficiently capture solar irradiation through a complex network of interconnected pores and protrusions containing black pigments (Cu_0.64_Cr_1.51_Mn_0.85_O_4_). These pigments were previously bonded by alumina (Al_2_O_3_, precursor: S75P) in the base layer and titania (TiO_2_, precursor: titanium(IV) isopropoxide; TTIP) in the absorption layer. A nanolayer containing silica nanoparticles bonded by a silica matrix^[^ ^11^ ^]^ acts as an antireflective top layer. The hierarchical architecture mainly enhances light trapping by promoting multiple internal reflections and increasing the optical path length within the coating structure, ultimately leading to improved absorption of solar irradiation. In terms of base and absorption layers, in this study two precursors were compared: tetraisopropyl isopropoxide (TTIP; also used in our previous study^[^ ^10^ ^]^) and titanium tetrabutoxide (TTNB). Although both precursors contribute to the formation of titania as the pigment inorganic binder, they differ significantly in their chemical properties and reactivity. TTIP, which is more reactive and volatile,^[^ ^30^ ^]^ is known to produce coatings with fine structures and high porosity.^[^ ^10^ ^]^ However, this study found that TTIP was not suitable as the base layer precursor, which is directly deposited on a heated substrate because pyrolysis (thermal decomposition of the organic precursor with titanium into titania) occurred before the coating settled uniformly on the substrate. In contrast, TTNB offers slower reactivity when hydrolyzed,^[^ ^31^, ^32^ ^]^ allowing us to spray the titanium precursor uniformly onto the heated substrate. Furthermore, TTNB still offers controllable film thickness and morphology, similar to that in the case of TTIP. The choice between these precursors profoundly influences the final properties of the coral‐structured coating, including its optical performance and morphology (micropores and macroscale protrusions). Thus, understanding the differences between TTIP and TTNB is crucial to optimize the coating performance.

**Figure 2:**
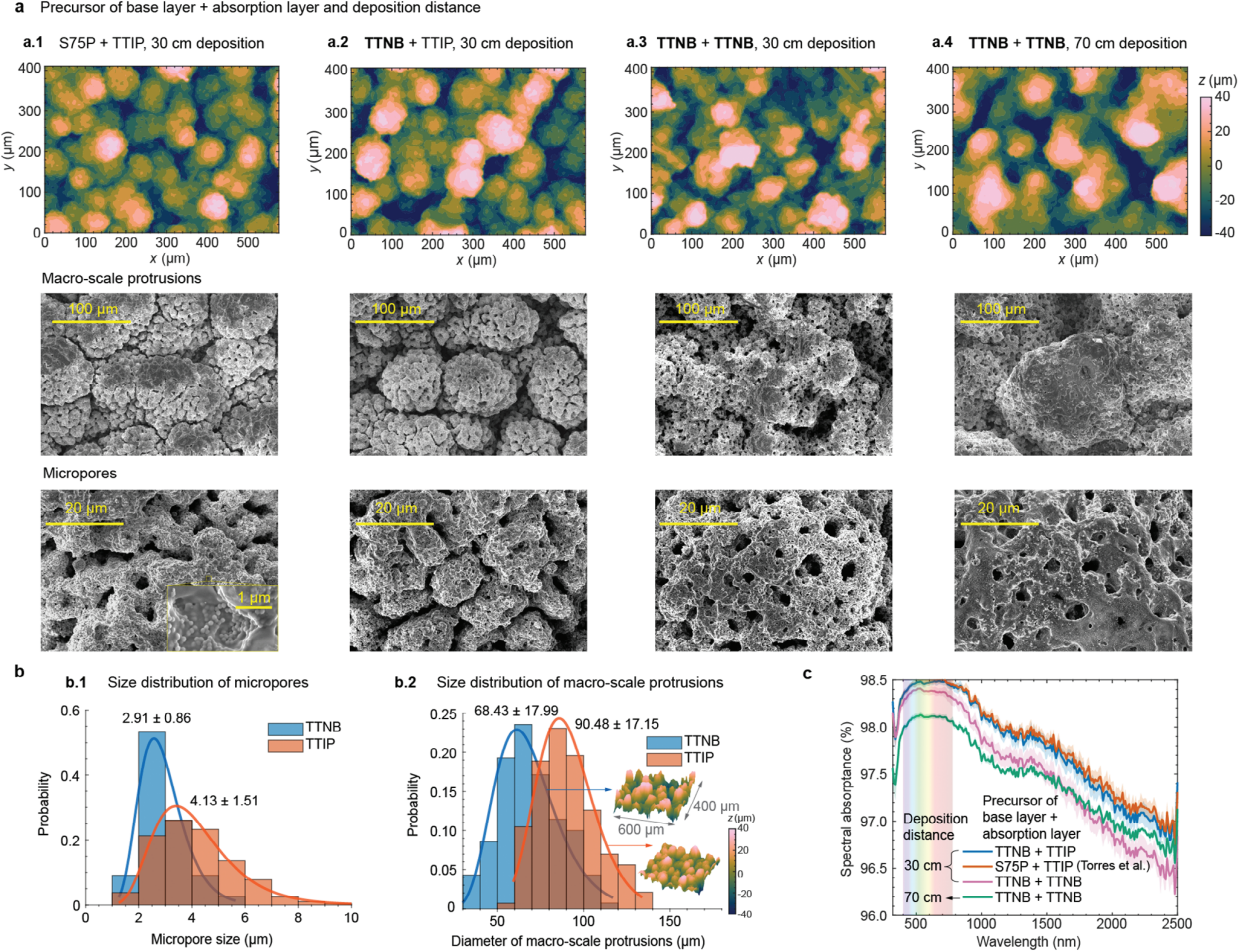
Morphology and absorptance of pristine coral‐structured coatings produced with different precursors. In this figure, S75P is an aluminum complex (aluminum ethylaceto acetate di iso‐propirate), TTIP is titanium isopropoxide and TTNB is titanium tetrabutoxide. a) Surface morphology (confocal microscopy and SEM images) of pristine samples when formed with different titania precursors in the base and absorption layers, as well as different spray distances. b) Histogram of size distribution of b.1)  micropores and b.2) macroscale protrusions in the absorption layer. The insets are 3D view of macroscale protrusions for the different precursors from the confocal microscopy measurement. c) Spectral absorptance of pristine samples.

When different precursors of the inorganic binder and spray parameters are employed in the fabrication process, the resulting morphology of the coral‐structured coating varies significantly, as shown by the confocal microscope measurements and SEM images in **Figure** **2**a. In particular, regardless of the base layer, the absorption layer using the TTIP precursor exhibits a consistent morphology, as observed when comparing Figure 2a.1 and a.2. SEM analysis reveals distinct characteristics in both macroscale protrusions and micropores between coatings produced from TTIP and TTNB precursors, as depicted in Figure 2a.1–4. The size distribution in Figure 2b.1–2 quantifies these differences in morphology with a fixed deposition distance of 30 cm (from the nozzle to the substrate). The micropores produced with the TTNB precursor exhibit a slightly smaller size compared to those generated with TTIP, noting that the spray‐deposition conditions also varied, i.e., a similar morphology could be achieved in principle if the optimum spray‐deposition conditions are found (e.g., spray pressure, distance of nozzle tip to surface, etc.). Similarly, the macroscale protrusions are also smaller when TTNB is used as the precursor under current deposition conditions. These results highlight slight differences in morphology (both in micro‐ and macroscales) in the coral‐structured coatings, highlighting the need for optimization to achieve desired morphological characteristics that improve solar absorptance. A larger size and closer connection between pores can be seen when using the TTIP precursor. The arrangement and connectivity of pores within the coating can influence the path of light through the material. The highly interconnected pore structure and larger size allow for multiple light scattering events, which increases the light absorption within the material.

The spectral absorptance of coral‐structured coatings exhibits differences depending on the choice of precursor and deposition parameters, as indicated in Figure 2c. In pristine condition, the base layer formed from either S75P or TTNB had a marginal influence on the spectral solar absorptance for absorption layers produced with the TTIP precursor. In contrast, coatings based on the TTNB precursor for the absorption layer exhibited a lower absorptance, particularly in the infrared wavelength range. This difference in absorptance can be attributed to the morphological changes observed under the SEM (Figure 2b), specifically, the smaller size of the micropores and the macroscale protrusions. Moreover, the deposition distance is one of the parameters that can affect the morphology of macroscale protrusions (Figure 2a.4). In our experiments, a greater distance led to a reduction in spectral absorptance, particularly in the visible wavelength range, as shown in Figure 2c. However, this may not always be the case as other deposition parameters also affect morphology.^[^ ^10^ ^]^ The lower number density of macroscale protrusions, as indicated in the confocal microscope images of Figure 2a.3–4, could have decreased the number of multiple internal reflections and reduced the optical path length within the coating. Although a larger deposition distance lowered the absorptance here, it is more amenable to adoption in the industry because the receiver has a high‐temperature surface that may heat up the nozzle, affecting its operation. A larger deposition distance mitigates this risk.

### 2.2 Durability of Titania‐Based Coral‐Structured Coating

**Figure 3:**
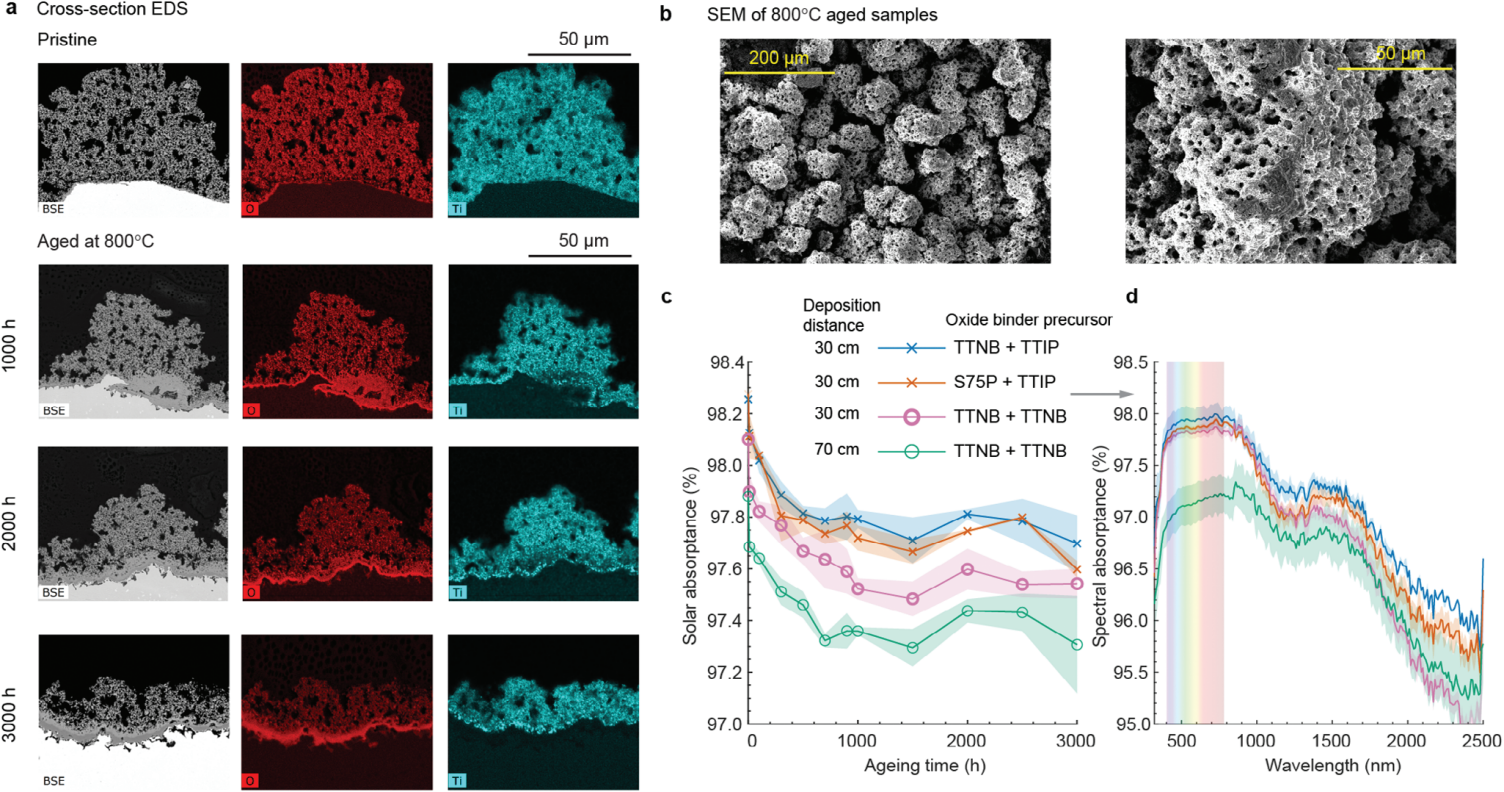
Confirmation for hindering cation diffusion and stability of titania‐based coral‐structured coating. Characterization of aged samples for samples produced with different titania precursors on bare Inconel. a) Cross‐sectional EDS of titanium in coral‐structured coatings for the base layer and absorption layer is TTNB with the deposition distance of 30 cm. b) SEM showing morphology of TTNB + TTNB after aging at 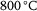. c) Solar absorptance as a function of aging time for different materials of coral‐structured coatings when aging at 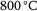. d) Spectral absorptance of four coatings after aging at 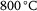 for 3000 h.

Sample analysis after extensive isothermal aging at 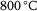 for 3000 h has confirmed the effectiveness of hindering cation diffusion in coatings produced with a base layer from TTNB precursors. The cross‐sectional EDS results in **Figure** **3**a unequivocally demonstrate the absence of titanium diffusion into the substrate after prolonged thermal aging. This result is in stark contrast to the previous base layer formulation using alumina as the inorganic binder of black pigments (Figure 1a), underscoring the significant impact of the oxide binder on interfacial interactions and coating durability. Inhibition of diffusion of ions can mitigate interfacial delamination, a common failure mechanism in coatings subjected to thermal stress.^[^ ^8^ ^]^ By preventing the migration of titanium cations into the substrate, the coatings with the base layer produced from the TTNB precursor exhibited interfacial stability with expected enhanced adhesion, thereby mitigating the risk of delamination and improving overall durability. This finding not only validates the efficacy of TTNB‐based base and absorption layers but also underscores the importance of considering diffusion phenomena in the design and optimization of protective coatings for high‐temperature applications.

The durability of coatings produced from TTNB precursors has been further confirmed by aging at 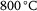 for 3000 h. SEM analysis reveals marginal changes in both macroscale protrusions and micropores after aging, as evidenced by comparisons between Figures 2a and 3b. Furthermore, a comparative assessment of solar absorptance in pristine condition shows a minimal disparity, with differences of less than 0.2% observed between coral‐structured coatings produced with TTNB and TTIP absorption layers (at the same spray distance). This marginal difference in solar absorptance persists even after aging, with statistically identical variations, as shown in Figure 3c. Upon hydrolysis and condensation, TTNB forms a dense and interconnected TiO_2_ network. This oxide layer acts as a barrier, limiting the mobility of cations within the coating matrix. The dense structure of the TiO_2_ network restricts the pathways for the movement of cations, thus reducing the diffusion rates. The TTNB‐based coating forms strong chemical bonds with the underlying substrate, further stabilizing the coating structure. This strong adhesion minimizes defects or “weak spots” where cation diffusion might otherwise occur more readily during the aging process. In addition, the TTNB‐based coating typically has fewer pore–oxide boundaries compared to that of other precursors. By reducing the surface area, the TTNB‐based coating might reduce oxidization. Spectral absorptance measurements in Figure 3d, after 3000 h of aging further support the nearly identical light‐trapping properties obtained for coatings produced from TTNB or TTIP precursors, particularly in the visible wavelength range. This similarity in spectral absorptance, especially after aging, presents a significant advantage, which might be attributed to the effective reduction of cation diffusion in TTNB‐based coatings. Using the same coating material (with TTNB as oxide binder precursor) in both base and absorption layers simplifies material preparation and decreases the complexity. These results collectively show the robust durability and stability of TTNB‐derived coral‐structured coatings, positioning them as promising candidates for high‐temperature applications requiring long‐term performance reliability.

### 2.3 Drone‐Deposited Coral‐Structured Coatings as Means of Repair and Maintenance of Solar Absorbers

**Figure 4:**
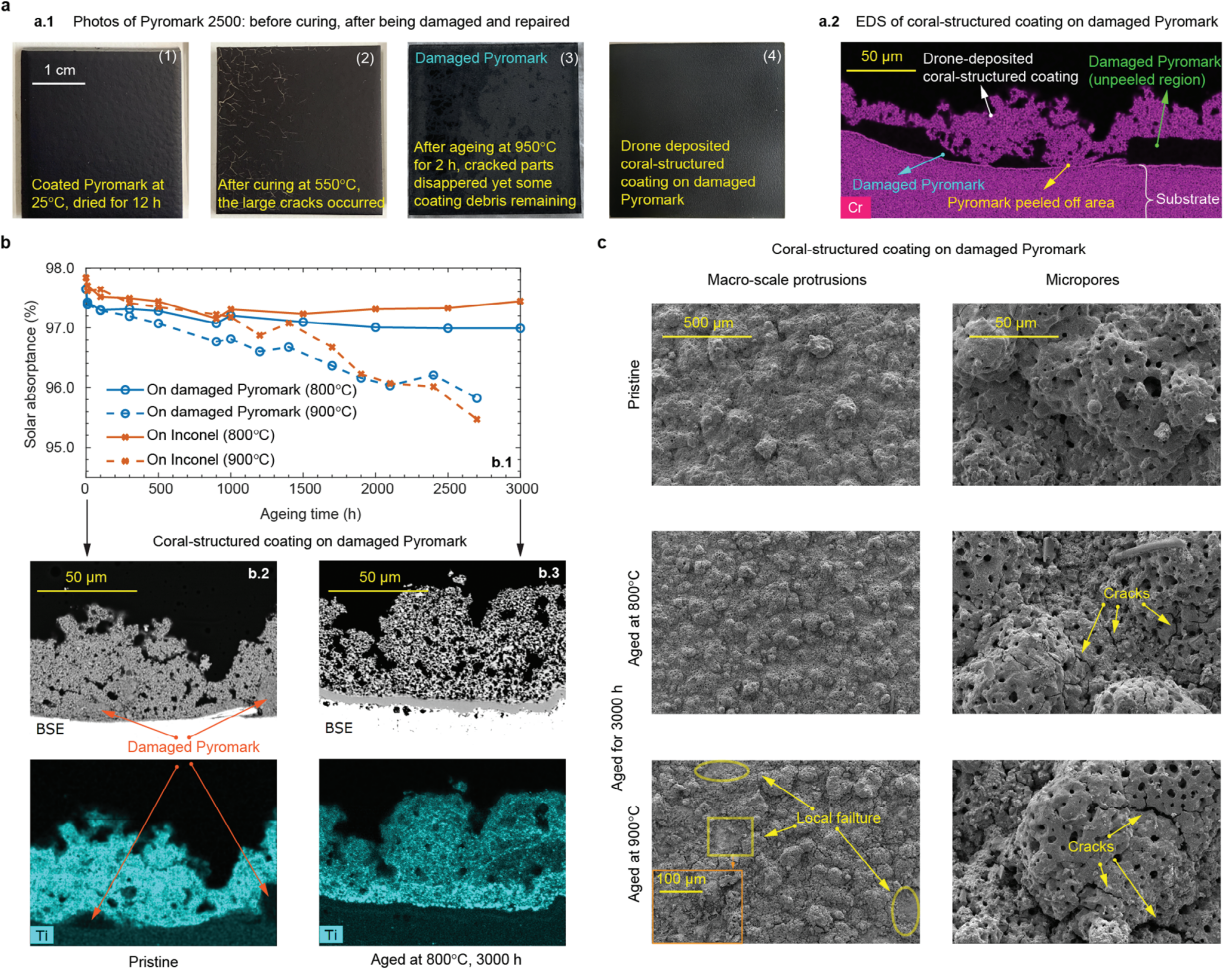
Coral‐structured coating as means of repair for damaged solar absorbers. a) Drone‐deposited coatings on damaged Pyromark. a.1)  Photo of damaged Pyromark and repair by drone‐deposited coral‐structured coating a.2) Cross‐sectional EDS of chromium indicating the component when drone‐deposited coral‐structured coatings on damaged Pyromark in pristine condition. b) Temperature dependence of drone‐deposited coatings. b.1) Solar absorptance as the function of aging time for drone‐deposited coral‐structured coatings on damaged Pyromark and Inconel with two different temperatures, i.e., 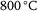 and 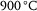. b.2) Cross‐sectional EDS showing drone‐deposited coatings on damaged Pyromark in pristine condition. b.3) Cross‐sectional EDS showing drone‐deposited coatings on damaged Pyromark when aging at 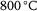 for 3000 h. c) SEM images of drone‐deposited coral‐structured samples on damaged Pyromark when aging at 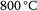 and 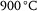 for 3000 h.

The introduction of drone‐deposited coating as a potential repair and maintenance approach offers promising prospects. The enhancement in solar absorptance and durability was assessed after applying the coral‐structured coating to degraded Pyromark samples (cured at the final temperature of 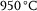, see detailed preparation in our previous study^[^ ^2^ ^]^) or damaged Pyromark coatings that experienced delamination. The damaged Pyromark coatings were prepared through a standard curing and aging process (see details in Experimental Section) that resulted in the appearance of cracks and peeling for large spallation regions, as shown in **Figure** **4**a.1(1)–(3). Subsequently, drone‐deposited coral‐structured coatings were applied, completely covering the damaged areas consisting of the oxide layer of the substrate and the degraded Pyromark, producing the black coating shown in Figure 4a.1(4). Cross‐sectional EDS analysis of chromium provided insight into the adhesion of the coral‐structured coating, suggesting a robust attachment to both the damaged Pyromark coating and the underlying substrate (thin layer of chromium oxide on Inconel) regions, as depicted in Figure 4a.2. Note that there is no chromium in Pyromark,^[^ ^8^ ^]^ which corresponds to the sandwiched region between the coral‐structured coating and Inconel. The cross‐sectional view in Figure 4a.2 reveals that the damaged Pyromark has some regions with uniform thickness (shown on the right), while some damaged Pyromark regions have irregular thickness (shown on the left). The large peeled area was covered by the coral‐structured coating. The good adhesion between the coral‐structured coating and damaged underlying materials reveals the possibility of applying drone‐deposited coatings as a viable approach to repair damaged solar absorbers, with the potential to enhance both performance and longevity in real‐world applications.

The Pyromark repaired with the drone‐deposited coral‐structured coating experienced marginal changes in solar absorptance after aging, as shown in Figure 4b.1, demonstrating the resilience of this coating repair and maintenance approach even after aging at high temperature for up to 3000 h. Initially, in pristine condition, the solar absorptance of the drone‐deposited coating closely matches that of the lab‐deposited coral‐structured coating on Inconel substrates with a deposition distance of 70 cm (Figure 4b.1 at *t* = 0), demonstrating consistency in performance across different deposition methods. The same trend was observed for Inconel or damaged Pyromark as underlying substrates: aging at 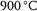 resulted in a downward trend in solar absorptance, while stability was maintained at 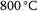. The thermal barrier at different locations is expected to change because there are peeled‐off areas and a remaining damaged Pyromark with variable thickness. Therefore, the heat that the coating is absorbing during deposition may vary locally, affecting its micro‐ and macroscale morphologies, which may be less optimal for solar absorption than the case of depositing the coating on a bare Inconel substrate. The absence of Ti^4+^ traces in the substrate after aging (Figure 4b.2,3) provides compelling evidence that cation diffusion did not occur, in stark contrast to previous results where aluminum diffused through the substrate (Figure 1a).

The characterization of the repaired Pyromark samples after aging revealed degradation due to occasional peeling at discrete locations and cracks, as shown in Figure 4c, similar to observations with coral‐structured coatings on Inconel substrates.^[^ ^10^ ^]^ However, the cracks observed on the coral‐structured coating on Inconel were much more severe than for the repaired Pyromark with the coral‐structured coating on top. This suggests that there is a mitigation of the thermal stress brought about by the degraded Pyromark. The degradation observed under the SEM can explain the drop in solar absorptance with aging. Interestingly, the morphological features in macroscale protrusions and micropores have minimal changes and are largely independent of aging temperatures, highlighting the robustness of the hierarchical coating structure against thermal stresses (Figure 4c). Few cracks were observed between the micropores under both 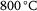 and 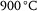 conditions, while few peeling regions were observed only at the temperature of 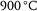. The unique coral‐like morphology, with its complex network of micropores and macroscale protrusions, may have regions where thermal stress is concentrated at higher temperatures. These stress concentration points can act as initiation sites for cracks when the material is subjected to thermal cycling or to prolonged exposure to high temperatures. The local failure observed at 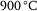 is probably due to the combination of increased crack formation and thermal stresses that exceed the adhesive strength of the coating. The mechanical resilience of coral structures can also create pathways for crack propagation that eventually lead to local failure. However, we note that the propagation of the local failure is very limited in coral‐structured coatings because of their discrete macro‐ and micro‐scale features. When failure occurs, local peeling occurs, but the entire coating does not delaminate as observed in Pyromark, a conventional CST coating.^[^ ^2^ ^]^ This explains the higher solar absorptance of the coral‐structured coating on the damaged Pyromark than that for the bare Inconel, after aging at 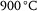 after 2000 h (Figure 4b.1). The resilience and stability of drone‐deposited coral‐structured coatings on damaged Pyromark substrates were confirmed under high‐temperature conditions, positioning them as promising candidates for applications in demanding industrial environments.

**Figure 5:**
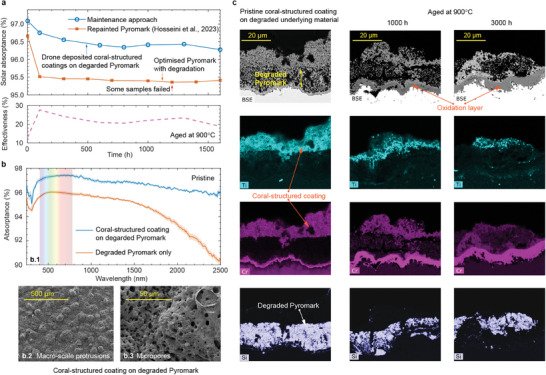
Coral‐structured coating as means of maintenance for degraded solar absorbers. a) Solar absorptance as the function of aging time for drone‐deposited coral‐structured coating on degraded Pyromark and optimised Pyromark on sandblasted Inconel when aging at 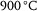. The effectiveness of this maintenance approach is shown in the lower plot. b) Characterization of coral‐structured coating on the degraded Pyromark in pristine condition. b.1) Spectral absorptance of degraded Pyromark with and without coral‐structured coating. b.2) SEM image showing the macroscale protrusions. b.3) SEM image showing the morphology of the micropores. c) Cross‐sectional EDS of our drone‐deposited coating in the degraded Pyromark in pristine condition and after aging for 1000 h and 3000 h in 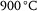.

The use of drone‐deposited coatings may present a viable maintenance approach when applied to degraded Pyromark coatings, offering a standardized method for enhancing solar absorption by coral‐structured coatings. The degraded Pyromark was produced when cured at 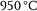, yielding a lower initial solar absorptance than the best performing Pyromark coating cured at 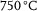.^[^ ^2^ ^]^ In conventional repair for the degraded Pyromark on the receiver, a common approach is to remove the degraded coating, sandblast the substrate, reapply the Pyromark coating, and then cure. Compared with maintenance by reapplying Pyromark on a sandblasted Inconel,^[^ ^2^ ^]^ the solar absorptance measurements in **Figure** **5**a revealed a substantial improvement in absorption after application of the coral‐structured coating on the degraded Pyromark, both just after maintenance and after extensive aging. Importantly, a 0.7% improvement in solar absorptance (absolute value) was observed after aging at 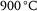 for 1600 h, while the optimized Pyromark begins to fail under such thermal conditions.^[^ ^2^ ^]^ The effectiveness *e*_c_ presents a metric to evaluate the enhancement in light trapping caused by the “maintenance” or “upgrade” method of depositing the coral‐structured coating on the degraded Pyromark. *e*_c_ is defined as

(1) $$e_{c}=\left(1-\frac{\rho_{c}}{\rho_{p}}\right)\times 100\%,$$

where *ρ*_c_ and *ρ*_p_ are the total hemispherical reflectance of the coral‐structured coatings on the degraded Pyromark (our proposed maintenance method) and the reflectance of the optimized Pyromark on the sandblasted Inconel (conventional maintenance method), respectively. *e*_c_ = 1 means that the “upgrade” method was absolutely efficient, producing a blackbody with zero reflectance, *ρ*_c_ = 0. The effectiveness of maintenance exceeds 20% after aging, even after severe thermal testing (Figure 5b.1, bottom plot). The coral‐structured coating on degraded Pyromark improved absorptance throughout the entire wavelength spectrum, as shown in Figure 5b.1 for the coating just after maintenance (pristine). An improvement in solar absorptance of 1.5% (absolute value) was observed. However, comparing the solar absorptance of the coral‐structured coating on the degraded Pyromark (for maintenance) and on damaged Pyromark (for repair), i.e., Figure 5a (top) versus Figure 4b.1, it was found that the solar absorptance in the pristine repair case was slightly lower (97.1% vs 97.7%), although the micro and macroscale morphologies are nearly identical (Figure 5b.2,3 vs Figure 4c). This discrepancy is attributed to the local variation in the thermal barrier caused by changes in thickness (within 20 µm) of the pre‐existing Pyromark coating. In the backscatter electron (BSE) images shown in Figure 5c, the layer with a high porosity represents the degraded Pyromark sandwiched between the coral‐structured coating (top) and Inconel (bottom). In particular, consistent with observations on bare substrates and damaged Pyromark, there was no evidence of Ti^4+^ diffusion from the coral‐structured coating to the underlying damaged Pyromark. EDS analysis elucidated the elemental composition of the components, with Si mostly present in Pyromark, and Cr present in the substrate and coral‐structured coating. During aging, there was a large growth of the oxide layer beneath the degraded Pyromark, with Cr^3+^ cations diffusing from both the coral‐structured coating and Inconel. The coral‐structured coating has demonstrated superior durability compared to the repainted Pyromark. However, whether it can serve as a potential replacement for Pyromark depends on several factors. Lowering materials and deposition costs can be achieved by standardizing industrial processes. Further exploration of economic potential, such as the levelized cost of energy (LCOE), is needed to fully assess the viability of the coral‐structured coating as an alternative to Pyromark. These findings underscore the efficacy of drone‐deposited coatings as a maintenance strategy to enhance solar absorption on degraded solar absorber coatings, while also providing valuable insight into the underlying mechanisms governing coating performance and durability.

### 2.4 Industrial Scalability of the Developed Coral‐Structured Coating

**Figure 6:**
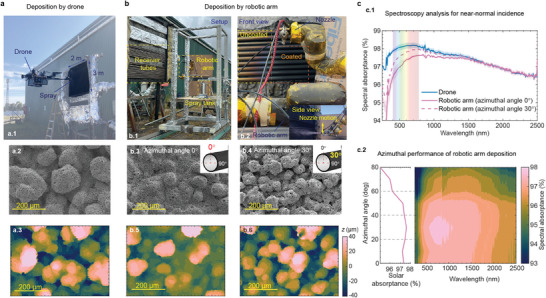
Scalability validation from on‐site deposited coral‐structured coatings. a) Deposition with drone. a.1) Photo of drone deposition with a scale of 2 m by 3 m receiver tube sets (20 tubes). a.2) SEM image of the coral‐structured coating obtained from drone‐assisted deposition on a full‐scale receiver. a.3) Confocal microscopy of the coral‐structured coating obtained from drone‐assisted deposition. b) Deposition with the robotic arm. b.1) Photo of robotic arm deposition setup. b.2) Photo of deposition by robotic arm; front view. The uncoated and coated regions can be clearly distinguished from their color; the dark black region is the coated region. The inset shows the side view with vertical motion of the nozzle driven by the robotic arm. b.3) SEM image of the coral‐structured coating through the on‐site deposition by the robotic arm for the azimuthal angle of zero. b.4) SEM image of the coral‐structured coating through the on‐site deposition by the robotic arm for the azimuthal angle (defined in the inset) of 30°. b.5–6) Confocal microscopy of the coral‐structured coating through the on‐site deposition by the robotic arm for the azimuthal angle of 0° (b.5) and 30° (b.6). c) Optical analysis of on‐site deposited coral‐structured coatings. c.1) Spectral absorptance of coral‐structured coatings with drone deposition and robotic arm deposition in pristine condition. The solid lines are the average spectral absorptance of four measured samples at different locations in the receiver tubes, and the shaded region represents the variation of ±0.05% at a confidence level of 90% for the two deposition methods. c.2) Azimuthal performance of the coating from robotic arm deposition.

To scale up coating processes onto large receiver surfaces, an in situ deposition was carried out Vast Energy (New South Wales, Australia). The coral‐structured coating was sprayed with two industrial methods, i.e., an unmanned aerial vehicle (UAV or drone) and a robotic arm, as shown in **Figure** **6**a.1,b.1, respectively (see Movie for the deposition process, Supporting Information). Drones excel in scenarios where repair and maintenance are required at much higher altitudes than 150 m, where access via traditional means may be limited. In contrast, when coating applications can be conducted at ground level, robotic arm deposition emerges as the most suitable option due to its scalability and precision. The use of a robotic arm eliminates the safety risks associated with operator exposure to high temperatures and toxic gases during the coating process. The precise control over coating parameters such as deposition distance, nozzle motion speed, coverage area, and number of passes are controlled via a pre‐set program. Photos in Figure 6b.2 indicate the precision of the robotic arm. The coated regions were applied uniformly and accurately without the need for manual adjustments.

The macroscale morphology shown in Figure 6a.2–3 and b.3–6 further confirmed the quality of the coral‐structured coating deposited using industrial methods. The macroscale protrusions of the coral‐structured coatings deposited by the drone were more uniform than when deposited by the robotic arm. Confocal microscopy indicates that the root mean square (RMS) average of profile height deviations was 21.66 µm for the drone, regardless of the azimuthal angles of the tubes (see inset of the Figure 6b.3 for azimuthal angle definition). In contrast, macroscale protrusions exhibited an azimuthal angle dependence for the robotic arm, as shown in Figure 6b.3–6. For example, a higher RMS value of 19.67 µm was observed for the azimuthal angle of 30° than that of 15.16 µm for the normal azimuthal angle (0°). Figure 6c further showed the spectral absorptance of the coral‐structured coatings deposited on site. This confirmed that the solar absorptance depends on the azimuthal angle for the robotic arm deposition. Specifically, the absorptance increases at azimuthal angles of 20–40°, as shown in Figure 6c.2. Unlike previous lab‐deposited coatings, where the curved coupons were placed with a spacing, the receiver tube has a cylindrical shape, and the on‐site deposition was conducted on closely positioned receiver tubes. The overspray appeared to spread more widely than intended for robotic arm deposition, leading to an oblique spray that generated more protrusions at azimuthal angles greater than 20° (with higher absorptance). In contrast, for drone deposition, the nozzle had a swinging motion that helped to create more uniform protrusions for a wider range of azimuthal angles. The nozzle movement differences can be found in the Movie (Supporting Information). The drone deposition achieved a spectral absorptance over 98% in the visible range, as shown in Figure 6c.1, indicating excellent absorption efficiency.

The scalability tests conducted in this study demonstrated the potential of robotic arm automation to scale up the coating deposition process. The use of a robotic arm resulted in substantial improvements in safety, accuracy, and repeatability. In contrast, drones face challenges in windy conditions, which may make them more sensitive to environmental constraints, while the ability to apply the coating at high altitudes is a strength, particularly for maintenance and repair. The results showed that the precise control of the robotic arm over movement and distance significantly improved the uniformity and quality of the coatings compared to those of manual deposition. To minimize the azimuthal dependence of the coating deposited by the robotic arm, an improvement could be adding another degree of freedom to the nozzle control such as a swinging motion (see the Experimental section for more details on the robotic arm deposition). The scalability of the process was evident as the robotic arm efficiently handled large surfaces without the need to dismantle the spray deposition system, thus saving time and reducing labor costs. In addition, the automation process improved safety by minimizing human exposure to hazardous conditions. The practical implications of this study suggest that robotic arm‐assisted deposition of the multi‐scale hierarchical coating is a process that could be widely adopted in various CST settings, offering a more efficient, safe, and scalable solution for deposition on large surfaces. Prospects include further advances in robotic programming and sensor integration to enhance the adaptability and precision of coating processes in even more complex environments.

## 3 Conclusion

Our investigation of the development and application of coral‐structured coatings with titania as the inorganic binder of black spinel pigments has yielded some key advancements and insights. Previous studies used alumina as the inorganic binder of the coral‐structured coating, but cation diffusion into the substrate affected the long‐term thermal stability of the coating. We have successfully demonstrated that cation diffusion into the substrate is effectively inhibited when titania is used as the inorganic binder (of black pigments) in the base layer. Our extensive aging results demonstrate that the coating has good stability and adheres well to the substrate. The lack of cation diffusion indicates a sustained bonding strength between the coating and the substrate even after aging, ensuring long‐term durability.

Moreover, our titania‐based coating exhibits long‐term optical and mechanical stability at high temperatures, making it a promising solution for various industrial applications. Furthermore, our coating presents a versatile approach for repairing, maintaining, or upgrading damaged or existing solar absorber coatings. Through drone and robotic arm‐based depositions, we have demonstrated the scalability of our coating onto solar thermal receivers, with each deposition method offering unique advantages depending on the application scenario. Optical characterization of the samples deposited in the field demonstrated the robustness of our coating in real industrial settings. Our titania‐based coral‐structured coating produced with TTNB as a titania precursor holds immense potential to improve solar thermal energy conversion in the concentrating solar thermal industry. The newly developed coating surpasses the durability and versatility of the previous alumina‐based coating, offering a reliable and scalable solution for high‐flux, high‐temperature solar thermal energy systems.

## 4 Experimental Section

### Precursor Preparation

The titanium tetrabutoxide (TTNB) precursor was prepared by mixing 1021 g of the titanium precursor titanium (IV) tetrabutoxide and 600.8 g of acetylacetone. A chemical reaction between those two materials occurred at 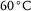 for 12 h. After the reaction, 35.5 g of ethylene glycol monobutyl ether was added and stirred at 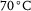 for 3 h. Then, polycondensation occurred after mixing 81.1 g of ion exchanged water and 138.2 g of ethanol with the above solution (stirring at 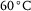) for 24 h. 882 g of diethyleneglycol isopropyl ether was added and stirred at 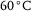 for 12 h. Lastly, 1182.2 g black pigments were added and stirred at room temperature for 3 h.

### Underlying Solar Absorber Preparation

The damaged Pyromark was prepared through a standard curing and aging process shown in Figure 4a.1(1)–(3). The Pyromark was first coated at room temperature (approximately 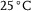) with a standard deposition method^[^ ^8^ ^]^ and dried for 24 h. The curing was then performed with multiple steps, starting at 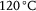 for 2 h, then 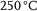 for 2 h and a final annealing for 1 h at 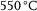. During this time, large cracks were found on the surface and a large peeled area appeared, as shown in Figure 4a.1(2). After continuing to age at 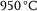 for 2 h, some coating debris remained (damaged Pyromark) and cracks were difficult to see with the naked eye because the Inconel substrate oxidized. In contrast, the degraded Pyromark was first sprayed on Inconel under the same condition as described by Torres et al.,^[^ ^10^ ^]^ and cured in the programmable muffle furnace at the maximum temperature of 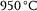. During the curing process, the following transit temperature was established (before high temperature annealing): 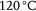 for 2 h, 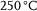 for 2 h and 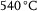 for 1 h, as described by Hosseini et al.^[^ ^2^ ^]^ The morphology in the degraded Pyromark experienced large crystal grain growth, which introduced a lower initial solar absorptance than the optimized Pyromark coating cured at 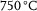.^[^ ^2^ ^]^

### Deposition Method

The substrate (Inconel) was first heated to the temperature of 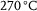, and the base layer was sprayed. Acetylacetone coordinated with titanium was slowly dissociated and evaporated, creating a flat coating with micropores. The porosity in the base layer helps the absorption layer above cope with the thermal expansion of the substrate. In contrast, acetylacetone coordinated with titanium is eliminated quickly and solidified by pyrolysis, forming titania almost instantaneously at the temperature of 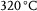. The surface temperature of the substrate was set to approximately 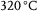 to form the absorption layer. After the macroscale protrusions were formed, the nanolayer was applied to the absorption layer, as described in our previous study.^[^ ^11^ ^]^

### Lab‐Based Deposition

The deposition was applied with Colani 2400 airbrush. For deposition of the S75P precursor for the base layer and TTIP for the absorption layer, the deposition condition is identical to that reported by Torres et al.,^[^ ^10^ ^]^ i.e., 22 spray passes for each layer. In contrast, less than ten spray passes were needed for each layer when coating with the TTNB precursor. The fewer spray passes reduced the risk of titania overgassing in adjacent regions and reduced the overall cost.

### Deposition by Drone

A custom‐made drone was used for deposition (Movie, Supporting Information). From the top view, the body size of the drone body was approximately 1.6 m × 1.6 m. To avoid disturbance from the drone propeller, a 1 m long nozzle was installed. The total weight of the drone was about 25kg. A specially designed camera and spray nozzle were attached to the drone. For a safe and accurate application of the coating, the drone was equipped with a distance sensor to maintain a distance of approximately 50 cm from the target while in flight. The spray nozzle was equipped with a two‐axis gimbal and a flight control system to perform tilt angle stabilization and spray trajectory angle error correction. As a result, even when the drone flight was disturbed due to wind, the distance from the spray nozzle to the target surface was kept nearly constant. In preliminary experiments (before the large‐scale receiver), a relatively small hotplate with dimensions of 50 cm × 50 cm was placed on a 1.8 m × 1.8 m backboard with 2 m above the ground (similar to that in ref. [11]). The plate was set at the required temperature for the corresponding layer (base, absorption, or nanolayer). The precursor solution, which was stored in the spray tank attached to the drone (Figure 1c.4), was then sprayed onto the samples. The coral‐like morphology formed after rapid evaporation (owing to the hot surface temperature) of acetylacetone coordinated with TTNB. In the Vast Energy concentrating solar thermal power plant in Jemalong, New South Wales, Australia, our custom‐made drone deposited the coating on the tube bank surface of a large‐scale receiver kept at a nearly isothermal surface temperature by circulating hot oil with the required temperature as indicated above for each layer.

### Deposition by Robotic Arm

The method of coating employed in this experiment involved the use of a robotic arm (model UFactory xArm5) with five degrees of freedom shown in Figure 6b.2. The robotic arm has a payload capacity of 3kg and a maximum reach of 700 mm. Using this robotic arm allows the coating process to reach the repeatability of ± 0.1 mm. For this scale‐up experiment conducted at the Vast Energy solar thermal power plant, the robotic arm deposited the coating on the tube bank surface of a large‐scale receiver kept at an isothermal temperature by circulating hot oil. The receiver target area assigned for this experiment had a width of 2 m and a height of 1.5 m. In order to maintain a smooth and continuous coating of the target area, the robotic arm was installed on the mobile aluminum rig shown in Figure 6b.1 (setup). This rig moved along a rail installed in front of the receiver to expand the reach length of the robotic arm. The motion of the nozzle installed on the head of the nozzle was programmed and synchronized with the lateral movement of the mobile rig. The spray nozzle was programmed to move vertically to cover the height of the target area and then horizontally in segments of 12 cm. This process continued until the entire receiver was coated. The distance of 120 mm between each vertical coating created an overlap of 30 mm between the vertical coating bands. This distance was determined through multiple tests to ensure sufficient overlap between each vertical coating band. The coating process was meticulously structured into three parts to ensure optimal performance and durability of the coatings. Initially, the base layer was applied to the preheated tubes at approximately 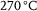. The absorption layer and the top (nano‐) layers were then applied with the tube temperature kept at 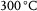. Because of the large thermal mass of the hot oil, the surface temperature during deposition was found to be very stable. The robotic arm applied the coating with a consistent vertical motion from bottom to top (as shown in the Movie, Supporting Information), maintaining a set speed and constant distance from the tube surfaces. The vertical speed of the spray nozzle for the base layer, the absorption layer and the top layer coatings are set in the range 10–15 mm s^−1^.

### Aging and Characterization

The isothermal aging was conducted in a programmable muffle furnace (LABEC SF–13–SD) with a convective heating rate of 3 K min^−1^ from ambient to the target temperature of 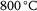 or 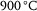. The furnace cooled down with a ca. 4 K min^−1^ after aging. The spectral reflectance (*ρ*(*λ*)) ranging from 250‐2500 nm was measured through a Perkin Elmer UV/VIS/NIR Lambda 1050 spectrophotometer with a 150 nm InGaAs integrating sphere (constant angle of incidence, 8°). Spectral absorptance measurements of lab‐based samples are for a near‐normal angle of incidence, i.e., the coating covers an aperture of the integrating sphere and the angle of incidence is 8°. Field samples were obtained from coated tubes on the receiver from which tubes of 1.5 m length were cut. Then, smaller tube samples of 3 cm in length were cut to fit inside the integrating sphere of the spectrophotometer. The spectral absorptance was measured while placing the coated tubes at the center of the integrating sphere, with one of its apertures sealed using the white standard (the other aperture is for the incident light) and the coated section of the tube facing the incident light. There was a marginal difference in the measured absorptance of the coatings (on flat coupons) between the placement on the aperture and inside the integrating sphere. Corrections of spectral reflectance measurements were made based on benchmark samples to increase the accuracy of this measurement (as described in ref. [10]). The spectral absorptance was calculated using *α*(*λ*) = 1 − *ρ*(*λ*), as there is no transmission. Solar absorptance can be calculated from the solar irradiance *G*(*λ*) standard of ASTM G‐173:

(2) $$\alpha =\frac{\int_{\text{280 nm}}^{\text{2500 nm}}\alpha \left(\lambda \right)G\left(\lambda \right)\;d\lambda }{\int_{\text{280 nm}}^{\text{2500 nm}}G\left(\lambda \right)\;d\lambda }$$

Scanning electron microscopy (SEM, Zeiss UltraPlus) and energy‐dispersive X‐ray spectroscopy (EDS, FEI Quanta QEMSCAN) were used to characterize the morphology and degradation of coating materials.

### Statistical Analysis

Unless specified, data were plotted as mean ± SD. Student's *t*‐test was applied for statistical tests and a *p*‐value < 0.1 was considered significant. For the solar absorption degradation experiment, four samples for each condition were characterized. The size distribution of micropores and macroscale protrusions was analyzed through 120 features in the SEM images.

## Conflict of Interest

The authors declare no conflict of interest.

## Supporting information

Supplemental Movie 1

## Data Availability

The data that support the findings of this study are available from the corresponding author upon reasonable request.
